# Porous Biochar Supported Transition Metal Phosphide Catalysts for Hydrocracking of Palm Oil to Bio-Jet Fuel

**DOI:** 10.3390/ma15196584

**Published:** 2022-09-22

**Authors:** Napat Kaewtrakulchai, Araya Smuthkochorn, Kanit Manatura, Gasidit Panomsuwan, Masayoshi Fuji, Apiluck Eiad-Ua

**Affiliations:** 1 Kasetsart Agricultural and Agro-Industrial Product Improvement Institute, Kasetsart University, Bangkok 10900, Thailand; 2 College of Material Innovation and Technology, King Mongkut’s Institute of Technology Ladkrabang, Bangkok 10520, Thailand; 3 Center of Excellence in Particle Technology and Material Processing, Department of Chemical Engineering, Faculty of Engineering, Chulalongkorn University, Bangkok 10330, Thailand; 4 Department of Mechanical Engineering, Faculty of Engineering, Kasetsart University Khamphaeng Saen Campus, Nakhon Pathom 73140, Thailand; 5 Department of Materials Engineering, Faculty of Engineering, Kasetsart University, Bangkok 10900, Thailand; 6 Advanced Ceramic Center, Nagoya Institute of Technology, Tajimi 507-0071, Japan

**Keywords:** porous biochar, metal phosphide, palm olein oil, bio-jet fuel, hydrocracking

## Abstract

The upgrading of plant-based oils to liquid transportation fuels through the hydrotreating process has become the most attractive and promising technical pathway for producing biofuels. This work produced bio-jet fuel (C_9_–C_14_ hydrocarbons) from palm olein oil through hydrocracking over varied metal phosphide supported on porous biochar catalysts. Relative metal phosphide catalysts were investigated for the highest performance for bio-jet fuel production. The palm oil’s fiber-derived porous biochar (PFC) revealed its high potential as a catalyst supporter. A series of PFC-supported cobalt, nickel, iron, and molybdenum metal phosphides (Co-P/PFC, Ni-P/PFC, Fe-P/PFC, and Mo-P/PFC) catalysts with a metal-loading content of 10 wt.% were synthesized by wet-impregnation and a reduction process. The performance of the prepared catalysts was tested for palm oil hydrocracking in a trickle-bed continuous flow reactor under fixed conditions; a reaction temperature of 420 °C, LHSV of 1 h^−1^, and H_2_ pressure of 50 bar was found. The Fe-P/PFC catalyst represented the highest hydrocracking performance based on 100% conversion with 94.6% bio-jet selectivity due to its higher active phase dispersion along with high acidity, which is higher than other synthesized catalysts. Moreover, the Fe-P/PFC catalyst was found to be the most selective to C_9_ (35.4%) and C_10_ (37.6%) hydrocarbons.

## 1. Introduction

Over the last decade, the supply of commercial transportation fuels is likely to grow due to its continued economic expansion. The accompanying released greenhouse gas (GHGs) emissions caused serious environmental problems, especially air pollution, aggravated by the combustion of fossil fuels from the transportation sector. The consumption of aviation fuel or jet fuel used for aircraft, as most commercial jet fuels are produced from petroleum refining, follows the international standard specifications and has increased significantly because of growth in air transportation. The use of jet fuel obviously led to more air pollutants [1,2]. To alleviate these problems, the new generation of alternative transportation fuels (e.g., bio-gasoline, bio-jet fuel and green diesel) produced from the catalytic hydrotreatment of triglyceride-based oils became an area of interest due to its potential for environmental and economic benefits [3].

Commercial jet fuels are mainly composed of paraffin, aromatics, cyclic alkanes and iso-alkanes, specifically ranging from the C_9_ to C_14_ hydrocarbons [4]. However, triglyceride-based oils are refined to eliminate undesirable molecules to improve their properties to meet the jet fuel standardized specifications. Triglyceride transformations to renewable jet fuel involve the formation of n-alkanes through the deoxygenation of free fatty acids, which cracked from the hydrogenation and hydrogenolysis of triglycerides, followed by the isomerization and cracking of n-alkanes to produce lighter molecule hydrocarbons in the jet fuel range. As mentioned above, research on catalysis performance, oil feed with superior fatty acid profiles, and variables used in the upgrading process are strongly necessary and remain challenging in producing renewable jet fuel [5,6].

Technically, porous material-supported metal catalysts have been widely used for hydrotreating processes in many refineries. It is a promising technology to use for biofuel production. A catalyst with high acidity and commonly used acidic supports (e.g., zeolites and Al_2_O_3_) are more challenging to implement in the production of biofuels since it could exhibit high catalytic activity towards hydrocracking for the distillation of bio-jet fuel. However, the utilization of acidic support presented some drawbacks, including catalyst deactivation contributing to coke formation on metal products [7,8,9]. On the other hand, the acid sites of catalysts remain important for C–O and C–C bond cleavage during the deoxygenation step, cracking reaction, and isomerization of light molecule hydrocarbons in bio-jet production. 

Several recent studies have focused on producing bio-jet fuel by using supported metal and metal sulfide catalysts. Wang and co-workers [10] reported the production of bio-jet fuel produced from soybean oil using ruthenium supported by ZSM-5 compared to a commercial NiMoS_2_ catalyst. The highest yield of jet fuel, approximately 21%, was obtained from Ru/ZSM-5 catalysts. Moreover, the research from Kim and co-workers [11] demonstrated that the hydrocracking of triglycerides from palm oil to produce renewable jet fuel was conducted over Pt/synthesized zeolite and Pt/γ–Al_2_O_3_ catalysts. A Pt/ zeolite catalyst showed a significant imbalance between the metal and acid functions of zeolite in hydrocracking, indicating the quick catalyst deactivation by coking. 

In addition, the huge arenes formation and overcracking of the hydrocarbon product that occurred led to a reduction in the yield of the final bio-jet fuel. They claimed that the highest yield of bio-jet fuel, approximately 55%, was obtained under an optimized condition. Nonetheless, the investigation of a one-step process for renewable jet fuel from lipid compounds was reported. Zhang et al. [12] successfully developed a bio-jet fuel from waste cooking oil via hydrotreating with a hierarchical NiMo/USY@Al-SBA-15 zeolite catalyst. The optimal selectivity for a bio-jet fraction (C_9_–C_15_) was obtained at about 39.7% at a reaction temperature of 380 °C with excellent catalyst stability. They reported that a high acidity of catalyst would increase the selectivity of bio-jet fuel. In addition, the production of bio-jet fuel from palm oil through hydrocracking was compared by using different zeolites supported Ni nanoparticles (i.e., Ni/SAPO-34, Ni/MCM-41, Ni/HY, Ni/SAPO-11 and Ni/Hbeta). Similar conversions of palm oil to bio-jet fuel were obtained from various catalysts. In addition, the Ni/SAPO-34 catalyst exhibited the highest alkanes with low aromatic contents due to the high acid sites, and it was noted to be an excellent catalyst for the palm oil conversion to bio-jet fuel [13].

Recently, supported metal phosphide catalysts displayed excellent performance in HDN and HDS for any amount of time. Many studies revealed various chemical and biofuel productions with high reactivity and a long lifetime during the catalytic process, surpassing metal phosphide catalysts [14,15]. Zarchin et al. [16] investigated the performance of silica, and HY zeolite supported nickel phosphide catalyst on the hydrotreatment of soybean oil. They found that the Ni-P/HY exhibited a stable operation for more than 250 h. Several studies evaluated the production of biofuels via hydrotreating over supported metal phosphide catalysts from various model compounds such as lauric acid. These catalysts showed significant advantages, such as high reactivity and stability, without a sulfur-free compound. 

The various phases of metal phosphides supported on inert materials and acidic supports were tested during biofuel production through hydrotreating. Kim et al. [17] studied the effects of support materials, such as silica and ZSM-5, on beta and USY zeolites using Ni_2_P as the active phase on the hydrocracking of naphthalene into BTX (benzene, toluene, xylene) compounds. Their results showed that Ni_2_P/silica showed activity only for the hydrogenation of naphthalene. Conversely, the Ni_2_P supported on zeolites catalysts significantly displayed the reactivity in hydrocracking of BTX, in the following order: Ni_2_P/Beta > Ni_2_P/USY > Ni_2_P/ZSM-5 due to their acidity that could promote isomerization and cracking. However, porous carbons became an attractive support due to their advantages of high thermal stability, high surface area and good mechanical strength provided to the catalyst for use in various hydrotreating processes [18]. Liakakou et al. [19] reported the effect of activated carbon support on the hydrogenation of CO for the production of alcohols using a NiMo catalyst. The use of activated carbon as support increased the activity and selectivity compared to the unsupported catalyst. Furthermore, Cui et al. [20] revealed that the Cu supported on the activated carbon (AC) exhibited better catalytic performance than Cu/SiO_2_ in the hydrogenation of dimethyl oxalate to methyl glycolate. Excellent conversion and selectivity were achieved by using Cu/AC.

Conventionally, thermochemical conversions with activation, including chemical and physical activation, are applied for the production of porous carbon from several carbonaceous materials, such as agricultural wastes, organic wastes, and municipal solid wastes [21]. Nonetheless, local agricultural wastes can be used as alternative, renewable and sustainable resources for producing porous carbon [22].

Palm oil is an economical plant in Southeast Asia. According to food and agriculture statistics in 2021, almost 90 percent of palm oil supplied to global markets is produced from Indonesia, Malaysia, and Thailand. The number of oil palm plantations has more than doubled over the last decade and increased from 10 to 21 million hectares. The total crude palm oil produced is approximately 300 million tons [23]. After oil palm production (harvesting and processing), about 55% of palm fibers (i.e., empty fruit bunch, meso carp fiber, leaves, and male flowers) become solid waste when a ton of fresh fruit is processed [24]. These solid wastes left after the processing do not have a commercial use. Moreover, a large amount of palm oil can be used as a renewable feedstock for biofuel production. Hence, palm fiber (PF) becomes an effective raw feedstock for porous carbon production. To the best of our knowledge, no study has investigated the use of PF to develop porous biochar and applied it as a catalyst support. Consequently, the relevant agricultural waste, namely oil palm fiber, is attractive to develop as porous biochar for catalyst support in the hydrotreatment of triglyceride-based oils for producing biofuels.

In this study, we aimed to use the biomass potential of palm fiber for porous biochar as a catalyst supporter. The performance of PF porous biochar (PFC) supporting metal phosphide catalysts was investigated for catalytic hydrocracking palm olein oil to produce a bio-jet fuel using a continuous down-flow reactor. The reduction of the phosphate technique was used for the synthesis of metal phosphide catalysts. Then, the synthesized catalysts were characterized in terms of their physicochemical properties by using XRD, XPS, TEM-EDS, H_2_-TPR, NH_3_-TPD, and N_2_ sorption analysis. Moreover, we discuss the catalyst activity of metal phosphides on the product contributions were tested in the hydrocracking of palm olein oil.

## 2. Materials and Methods

### 2.1. Materials

Oil palm fibers were cut and sieved into a size between 0.5–3 mm to prepare porous biochar as a supporter of metal phosphide catalysts. Cobalt (II) nitrate hexahydrate (Co(NO_3_)_2_•6H_2_O, 98% purity), Nickel (II) nitrate hexahydrate (Ni(NO_3_)_2_•6H_2_O, 98% purity), Iron (III) nitrate hexahydrate (Fe(NO_3_)_2_•9H_2_O, 98% purity), and Ammonium molybdate tetrahydrate ((NH_4_)_6_Mo_7_O_24_•4H_2_O, 99% purity) were used as metal phosphide precursors (CARLO ERBA Reagents Co., Ltd., Paris, France).

Potassium hydroxide (KOH, 85% purity) and hydrochloric acid (HCl) were purchased from CARLO ERBA Reagents. High purity-grade (99.99%) nitrogen and hydrogen were used in these experiments. The palm oil feedstock (palm olein oil) was supplied from a local supermarket in Thailand. The fatty acid composition of the palm oil is mainly composed of lauric acid (C12:0) 0.4%; myristic acid (C14:0) 0.8%; palmitic acid (C16:0) 37.4%; palmitoleic acid (C16:1) 0.2%; stearic acid (C18:0) 3.6%; oleic acid (C18:1) 45.8%; linoleic acid (C18:2) 11.1%; linolemic acid (18:3), respectively [25].

### 2.2. Preparation of Supported Metal Phosphide Catalysts

**Figure 1:**
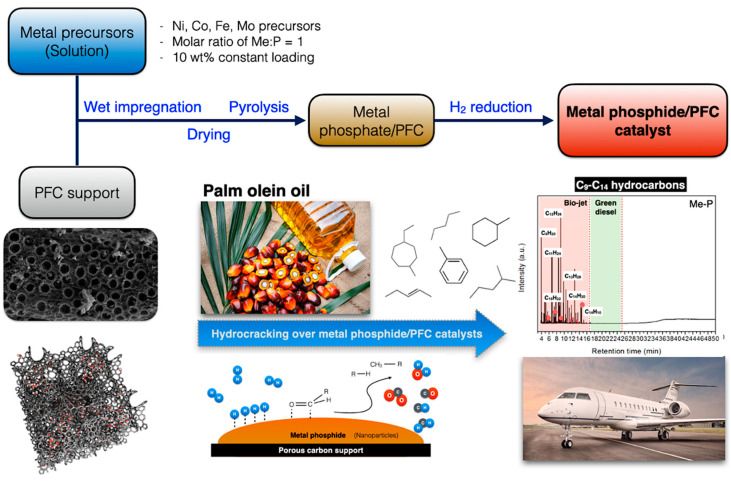
Process diagram of the synthesis of metal phosphide/palm fiber-activated biochar catalysts.

The PFC support was prepared from a microwave-assisted KOH activation—a method that has been reported elsewhere [26]. In the catalyst preparation, a series of corresponding phosphide catalysts were successfully synthesized via a wet impregnation technique with constant 10 wt.% metal loading followed by a reduction of metal phosphate compounds [27]. The catalysts were prepared by adding a stoichiometric amount of phosphoric acid (H_3_PO_4_ 99% CARLO ERBA Reagents) and the desired amount of relative metal transition precursors. The impregnated samples were dried in an electric oven at 80 °C overnight. Then, the dried samples were pyrolyzed at 800 °C for the Co- and Fe-based catalysts and 600 °C for the Ni- and Mo-based catalysts for 2 h to obtain the metal phosphate compounds. Subsequently, the phosphates were cooled to room temperature under an N_2_ stream and preserved for further catalytic testing. Before the test in the palm olein oil hydrocracking, reducing the metal phosphate into the corresponding metal phosphides under hydrogen is necessary to achieve the following metal phosphide/PFC catalysts, as illustrated in Figure 1. A high hydrogen flow rate was chosen due to its enhancement of water removal in the reduction process led to the formation of small metal phosphide particle sizes [28]. The reduction temperature followed the reducibility of each precursor conducted by H_2_-TPR analysis.

### 2.3. Catalyst Characterization

The textural pore characteristics were studied from the sorption isotherms of nitrogen at −196 °C carried out with a Quantachrome Autosorp iQ-MP-XR. The specific surface area was determined by using the BET model to the relative pressure (*P*/*P*_0_) below 0.3 by applying a value of 0.162 nm^2^ to the cross-section of an adsorbed nitrogen molecule in the calculation. Pore size distributions were explored by taking the DFT model to the N_2_ sorption isotherm. The total pore volume (V_T_) was evaluated by condensing liquid nitrogen at the relative pressure of 0.99 at the adsorption of nitrogen sorption isotherm.

X-ray photoelectron spectroscopy (XPS) of the as-prepared catalyst samples was conducted using Kratos AXIS Supra (XPS) surface analytical (Kratos analytical Ltd., Manchester, United Kingdom). Each sample was ground and reduced at 600 °C under H_2_ flow of 50 mL/min before the analysis; then, the reduced samples were situated on the carbon tape placed on the stainless-steel plate and substituted into a high vacuum system for measurement. The peak area was evaluated by calculating the integral of each peak after smoothing and subtracting an S-shaped background.

The studied catalysts’ dispersion and particle size were acquired by Transmission Electron Microscope (TEM), JOEL 2100 Plus (Akishima, Japan) at 200 keV. Before the observation, the sample was loaded onto a copper grid using an ethanol suspension.

The crystallinity and phase formation of the studied catalysts were conducted by X-ray diffractometer (Rikagu smart Lab, Tokyo, Japan) using Cu-Kα radiation (λ = 0.15418 nm), conducted at 40 kV and 40 mA, and in steps of 0.01° S^−1^ with a step time of 0.5 s over the range of Bragg angles between 10° and 80°. The crystalline sizes of samples were computed from the peak of the X-ray line width using the Debye–Scherrer equation. The width was taken as the full-width half-maximum intensity of the most intense and least overlapped peak.

NH_3_-temperature programmed desorption (TPD) was carried out using a Quantachrome Chemisorption Analyzer (ChemStar TPX Series, Quantachrome instruments, Boynton Beach, FL, USA). About 110 mg of samples was firstly reduced under H_2_ flow of 50 mL/min at 650 °C and then cooled to ambient temperature. Afterward, 5% NH_3_ in a helium flow of 30 mL/min was passed through the sample for 30 min at 100 °C, then switched to a helium flow of 30 mL/min to remove the physical adsorbed NH_3_ in the pretreatment step. Next, the NH_3_-TPD was conducted in a helium flow of 30 mL/min at a temperature from 100 °C to 600 °C using a heating rate of 10 °C/min. The desorbed NH_3_ was measured using a thermal conductivity detector (TCD).

H_2_-temperature programmed reduction (TPR) revealed the reduction characteristics of the studied catalysts. H_2_-TPR was studied using Quantachrome ChemBet Pulsar in a U-tube quartz reactor between 100 to 1000 °C with a heating rate of 10 °C/min under 5% H_2_/helium flow of 30 mL/min. A TCD detector detected the total H2 consumption.

### 2.4. Hydrocracking over Metal Phosphide/PFC Catalysts

A trickle-bed continuous flow reactor conducted the catalyst activity tests in hydrocracking. Typically, 8 mL of each catalyst (30–80 meshes) was packed in the middle of a stainless-steel tube reactor (I.D. 7 mm). The catalyst was “situ” activated by reduction at 600 °C for 3 h with a heating rate of 5 °C/min. After cooling to the desired reaction temperature of 420 °C, the palm olein oil was fed into the reactor system at LHSV of 1 h^−1^ using an H_2_ flow rate of 150 mL/min under H_2_ pressure of 50 bar. After the initial 4 h to achieve a steady-state reaction, the pretreated reaction liquid was drained. The liquid hydrocarbon product was collected after the reaction attained a steady state of a 2 h interval time for the analysis of offline gas chromatography (Shimadzu GC-MS QP2020, Kyoto, Japan) equipped with a capillary column (DB-1HT, 30 m × 0.32 mm × 0.1 μm) and a flame ionization detector (FID). Moreover, the gas products (CO, CO_2_, CH_4_, C_2_H_6_, C_3_H_8_, C_4,5_ gasses) were determined by online gas chromatography (Shimadzu GC-2010 Plus) with molecular sieve 5A and Porapak Q columns equipped with a thermal conductivity detector (TCD). The analysis results were directly computed to determine the palm oil conversion (Equation (1)), liquid hydrocarbon yield (Equation (2)), and bio-jet fuel selectivity (Equation (3)), respectively.

(1) $$\%\mathrm{Palm}\;\mathrm{oil}\;\mathrm{conversion}\;=\frac{\mathrm{Mass}\;\mathrm{of}\;\mathrm{palm}\;\mathrm{oil}\;\mathrm{feed}\;-\;\mathrm{Mass}\;\mathrm{of}\;\mathrm{palm}\;\mathrm{oil}\;\mathrm{in}\;\mathrm{product}}{\mathrm{Mass}\;\mathrm{of}\;\mathrm{palm}\;\mathrm{oil}\;\mathrm{feed}}\times 100\%$$

(2) $$\%\mathrm{Liquid}\;\mathrm{hydrocarbons}\;\mathrm{yield}\;=\frac{\mathrm{Mass}\;\mathrm{of}\;\mathrm{liquid}\;\mathrm{hydrocarbon}\;(C9\text{–}C24)\;}{\mathrm{Mass}\;\mathrm{of}\;\mathrm{palm}\;\mathrm{oil}\;\mathrm{feed}}\times 100\%$$

(3) $$\%\mathrm{Bio}-\mathrm{jet}\;\mathrm{selectivity}\;=\frac{\mathrm{Mass}\;\mathrm{of}\;\mathrm{hydrocarbon}\;(C9\text{–}C14)\;\mathrm{in}\;\mathrm{product}}{\mathrm{Mass}\;\mathrm{of}\;\mathrm{liquid}\;\mathrm{product}\;}\times 100\%$$

## 3. Results and Discussion

### 3.1. Characterization of Metal Phosphide Catalysts

**Figure 2:**
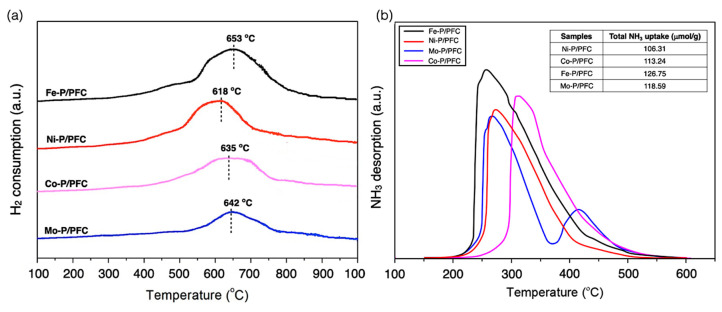
(**a**) H_2_ Temperature-programed reduction. (**b**) Temperature-programmed desorption of reduced catalysts.

Figure 2a illustrates the H_2_-TPR profile of the studied catalysts, which were provided to carry out the reduction ability of the synthesized catalysts. The H_2_-TPR profiles exhibited a character of a single peak at 550–700 °C that described the phosphate compounds reducing to metal phosphides. In addition, the total amount of H_2_ consumption of relative catalysts is listed in Table 1. The strong interaction between the metal species and carbon support surface was found in the broad peak of the H_2_-TPR profiles. The higher reduction temperature revealed an initial water loss peak between 550 and 650 °C, followed by a slowly developed broad feature with a high temperature at about 750 °C for all studied catalysts. The maximum reduction occurs at 600–700 °C. The reduction temperature of the obtained catalysts follows a trend: Fe-P > Co-P~Mo-P > Ni-P. Moreover, well-dispersed Metal-P particles revealed an increase in the amount of H_2_ consumption and the reduction temperature of catalysts (as seen in TEM images). This is because the small catalyst particles seemingly presented a higher interaction with the carbon surface [28,29].

Moreover, the acidity of the reduced catalysts was characterized by NH_3_-temperature programmed desorption (NH_3_-TPD). The NH_3_-TPD profiles and the total amount of adsorbed NH_3_ are demonstrated in Figure 2b. Regarding the NH_3_-TPD profiles, the studied catalysts showed a characteristic of medium and strong acid sites since the main desorption peak is centered approximately between 250–325 °C, with the NH_3_ desorption at a low temperature between 100 and 250 °C, which corresponds to a weak acidity (Brønsted acid site). Those at a higher temperature range (250–500 °C) are related to the moderate and strong acids (Lewis acid site) [30]. The P-OH groups in the phosphate species of an unreduced catalyst or M-OH groups were conducted at the range of weak acid site that corresponds to the Brønsted acidity. At the same time, the Lewis acid site and the moderate and strong acid sites came from the properties of some of the positive characteristics (M^δ+^) of the metal or metal phosphide particles due to the high electron mobility results from the crystal structure of the metal phosphide [19]. However, the total acidity of the studied catalysts was found to be as follows: Fe-P > Mo-P > Co-P > Ni-P. In addition, the total desorbed NH_3_ amount of supported Ni-P, Co-P, Fe-P and Mo-P catalysts were obtained at approximately 106.31, 113.24, 126.75 and 118.59 μmol/g, respectively. 

In this study, we found that the high acidic samples (Fe-P/PFC and Mo-P/PFC catalysts) presented the highest metal dispersion, as shown in the TEM image of studied catalysts. The higher acidity of Fe-P and Mo-P species may be exhibited from the Fe-OH and Mo-OH groups and phosphate species after the H_2_ reduction of catalysts. Furthermore, the strong acid sites are because of the metallic Fe and Mo sites, and the Fe and Mo phosphides have a greater positive charge than the Co phosphide and the Ni phosphide catalyst. Recent studies reported that the high acidity of catalysts assists the deoxygenation as presented in the cleavage of C-O bonds and hydrocracking, which is efficient for the formation of light hydrocarbons in renewable jet fuel production [16,31].

**Figure 3:**
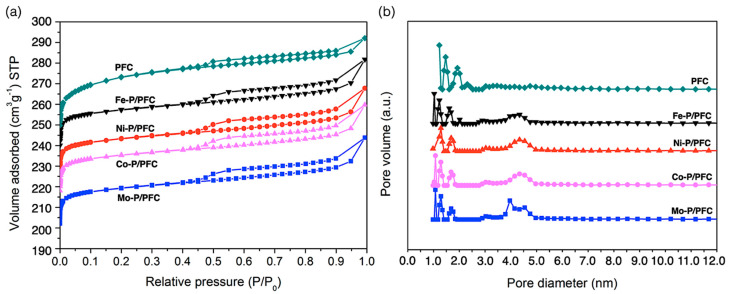
(**a**) Nitrogen sorption isotherms. (**b**) Pore size distribution obtained by applying the DFT method to the desorption step of PFC support and catalysts at −196 °C.

Figure 3 displays the textural pore structure analysis of the support and reduced catalysts. The characteristics of the Type I and Type IV adsorption isotherms with an H1 hysteresis loop were observed for all studied catalysts. This isotherm character is typical of porous materials combined with tiny mesoporous structures [18], as demonstrated in Figure 3a. Moreover, Figure 3b shows the pore size distributions of reduced catalysts and the produced carbon support. The PSDs of the studied catalysts were observed in two main distributions between 0.8 to 1.9 and 3.9 to 5.2 nm. The studied catalysts have narrow micropores ranges between 0.9–2.2 nm. On the other hand, the distribution of mesopores was only found on studied catalysts at approximately 2.7–5.1 nm. This is because the high temperature during catalyst preparation may significantly transform micropores to mesopores in bare carbon support [32].

As an observation in Table 1, all the studied catalysts showed similar textural properties, such as BET surface area, pore volume, and average pore diameter. The studied catalysts have a lower BET surface area and pore volume than the bare carbon support, suggesting a partial pore structure collapsing by high-temperature calcination and the incorporation of the active phosphide phase. The BET surface areas of the reduced catalysts are approximately between 629 and 792 m^2^/g.

**Figure 4:**
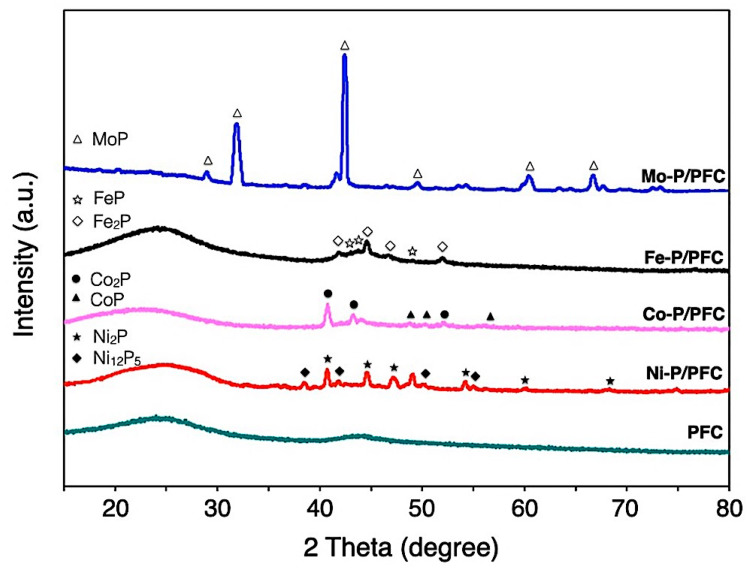
XRD patterns of reduced metal phosphide catalysts.

**Figure 5:**
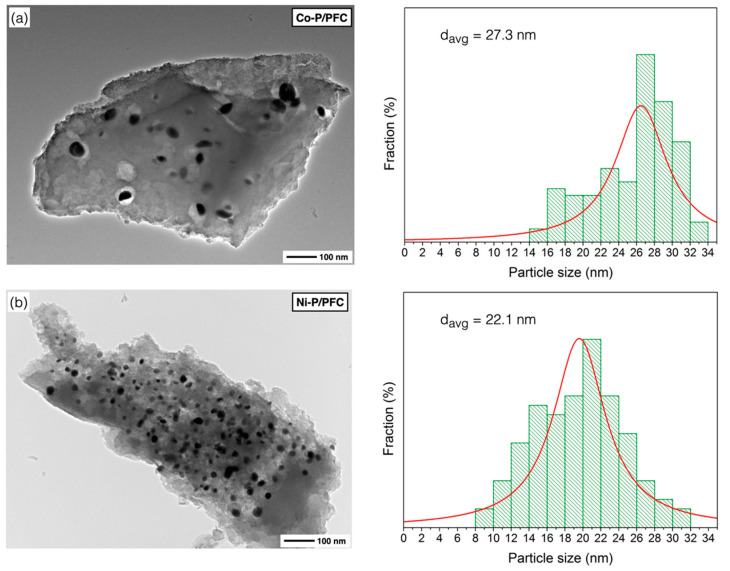

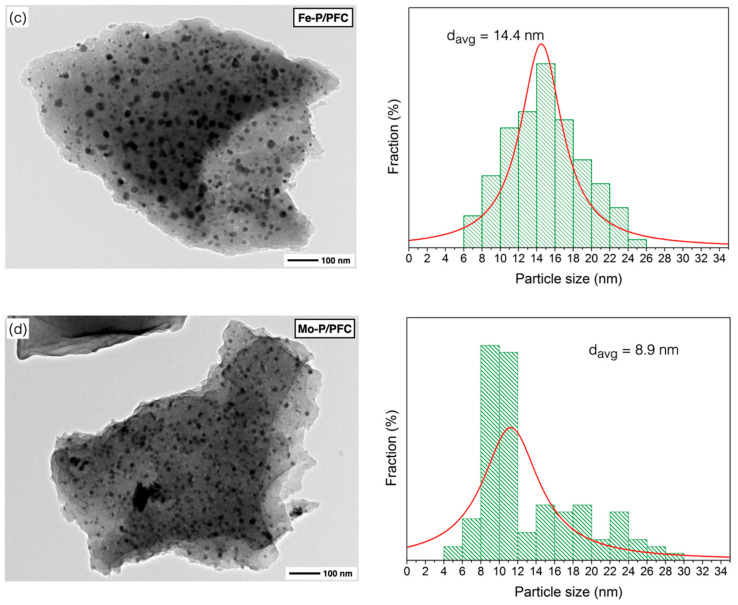
TEM images of reduced catalysts: (**a**) Co-P/PFC, (**b**) Ni-P/PFC, (**c**) Fe-P/PFC, and (**d**) Mo-P/PFC.

The XRD diffraction of the studied catalysts is represented in diffraction peaks, as seen in Figure 4. All XRD patterns show a spectrum at 15–35° of 2θ angle, which was the amorphous carbon diffraction [33]. Average metal-crystal sizes were determined by the Scherrer equation using XRD intensity of relative catalyst using all of the observed peaks from the analysis (labelled peak as seen in Figure 4), which are reported in Table 1. The domain sizes of catalysts followed the trend: Co-P > Ni-P > Mo-P~Fe-P, and the obtained values complied with the observation by high-resolution TEM, as illustrated in Figure 5. In the case of PFC support, the diffraction line is not observed due to the amorphous structure of carbon. Hence, it was impossible to determine this crystalline phase’s average domain size.

The Co-P/PFC catalyst revealed the diffraction contributions at 40.8, 43.4 and 52.1°, corresponding to the XRD pattern of the crystal planes of CoP (PDF 26-0481). However, the peaks centered at 48.3, 52.2 and 56.4° assigned to the Co_2_P formation (PDF 89-3030), which can observe in a tiny intensity [14]. The mixture of highly crystalline Co_2_P and CoP phases was found at high formation temperatures. Recent studies mentioned that CoP formed significantly at high temperatures, which requires a high phase formation energy. From these findings, it can be mentioned that increasing forming temperature during pyrolysis or calcination results in the transformation of Co_2_P to CoP [34,35]. In addition, the XRD pattern of supported nickel phosphide on PFC demonstrates the XRD intensity ranges of 35–75°. The diffraction lines were mainly Ni_2_P (PDF 65-1989), with low-intensity peaks corresponding to Ni_12_P_5_ (PDF 22-1190) [16,17]. The Mo-P/PFC catalyst presents higher intensity in XRD diffraction lines. The main species was the MoP phase (PDF 24-0771) [31]. In the Fe-P/PFC catalyst, the XRD patterns assigned to the FeP (PDF 81-1173) and Fe_2_P (PDF 85-1725) phases consist of overlapped diffraction lines between 40–55° [31,36]. This indicates that the Fe_2_P transformation to FeP is flawed during pyrolysis in the catalyst preparation.

According to the calculation of mean domain sizes, the average crystallite size of prepared phosphides showed the following trend: Co > Ni > Mo > Fe, as seen in Table 1. The average crystallite sizes of the studied catalysts also correlated with the results from the TEM observations. Moreover, phosphide particles can be accommodated on the surface of PFC support with good dispersion (Figure 5).

Figure 5 shows the TEM micrographs with the particle size distribution for the different studied catalyst samples. The micrographs agree with those of the metal phosphide catalysts studied by other authors [14,17,36], which are observed dispersed small particles on the support surface (porous biochar). Generally, metal phosphide particles are dispersed on the carbon matrix except on some sites that were impacted by a local agglomeration. 

Figure 5a presents the surface morphology of the Co-P/PFC catalyst, which contained poor catalyst particle dispersion due to metal aggregation. This indicated that a sufficient formation temperature was reached, and it may reach the transformation of Co_2_P to the CoP species from the harsh conditions during catalyst preparation [34,35]. In the case of the Ni-P/PFC catalyst, a mixture of Ni_2_P and Ni_12_P particles was observed on the carbon surface, which exhibits a spherical shape with high dispersion, as shown in Figure 5b, suggesting that the Ni species exhibited an excellent dispersion on different supports [35,37].

Moreover, the TEM image of Fe-P and Mo-P supported on PFC catalysts are displayed in Figure 5c,d. The particles of these two phosphide catalysts have a spherical shape with excellent dispersion on the carbon support surface. The highest dispersion of metal phosphide was observed on the Fe-P/PFC and Mo-P/PFC catalysts, respectively. From these findings, we could imply that the Fe-P/PFC and Mo-P/PFC catalysts could exhibit excellent hydrocracking activity and selectivity for bio-jet fuel production. The Fe-P and Mo-P nanoparticles were well-dispersed on the PFC surface compared to the Ni-P and Co-P catalysts. The average particle sizes and particle size distribution are also displayed in Figure 5. The Co-P, Ni-P, Fe-P and Mo-P nanoparticles have an average size between 8.9 and 27.3 nm. However, the particle size distribution of metal phosphide nanoparticles is correlated with crystallite size, as shown in the XRD analysis.

**Figure 6:**
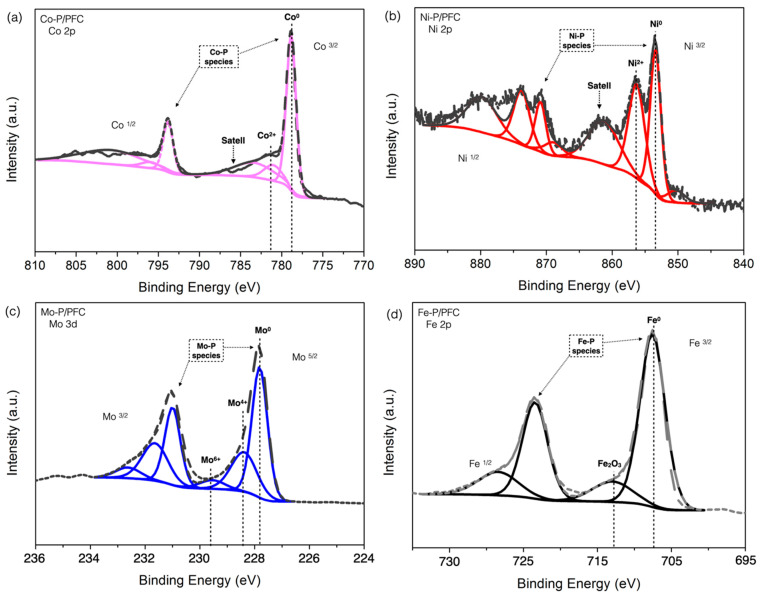
XPS of reduced catalysts: (**a**) Co-P/PFC, (**b**) Ni-P/PFC, (**c**) Mo-P/PFC, and (**d**) Fe-P/PFC.

Moreover, XPS spectra of reduced catalysts are displayed in Figure 6 with the binding energies of Co 2p_3/2_, Ni 2p_3/2_, Mo 2p_5/2_, and Fe 2p_3/2_ core levels. A sharp peak in Figure 6a and low binding energy at 778.3 eV is attributed to the Co^δ+^ species, represented in the phosphide s(Co_2_P or CoP phase). There is another peak at the higher binding energies located at approximately 782.0 eV, which is mainly attributed to unreduced species (Co^2+^ ions) from the phosphate and oxide groups, resulting from the incomplete reduction and surface oxidation during XPS sample preparation [17]. 

Figure 6b shows the Ni 2p spectrum of the Ni-P/PFC catalyst. The main contribution was separated into two contributions. The first is centered at 853.7 eV and is attributed to metallic nickel in phosphide. The second high-intensity spectrum was approximately 856.4 eV, accompanied by a broad satellite peak, which was assigned to Ni^2+^ in unreduced PO^3−^/PO^4−^ [16,17]. 

The XPS spectrum of the Mo-P/PMF catalyst is presented in Figure 6c. The different oxidation state of molybdenum (Mo 3d_5/2_) was presented in binding energy at approximately 227.8 eV, assigned to Mo^δ+^ site forming MoP. On the other hand, the peaks centered at 228.4 eV and 229.7 eV were assigned to partially reduced molybdenum, Mo^4+^ and Mo^6+^ species, respectively [31]. Similarly, the Fe 2p_3/2_ spectra of the reduced Fe-P/PFC catalyst are shown in two main contributions (Figure 6d). The first spectrum corresponded to the binding energy of metallic iron (Fe^δ+^) at a binding energy of 707 eV in iron phosphide structure (FeP and Fe_2_P sites) [31]. 

Furthermore, the binding energy centered at approximately 712.6 eV along with the satellite peak is ascribed to the oxide layer on the iron surface, Fe_2_O_3_ species [36]. From the XPS results, it can be concluded that the surface of the samples was partially reduced to the phosphide structure. The relative proportion of the unreduced phases in each catalyst is formed by oxidation of the catalyst surface during XPS sample preparation. This may affect by the incomplete reduction process. However, it can be implied that both the metal and phosphorous dispersions greatly depend on the transition metal types.

### 3.2. Hydrocracking of Palm Olein Oil

#### 3.2.1. Effects of Different Metal Phosphides on Bio-Jet Selectivity

**Figure 7:**
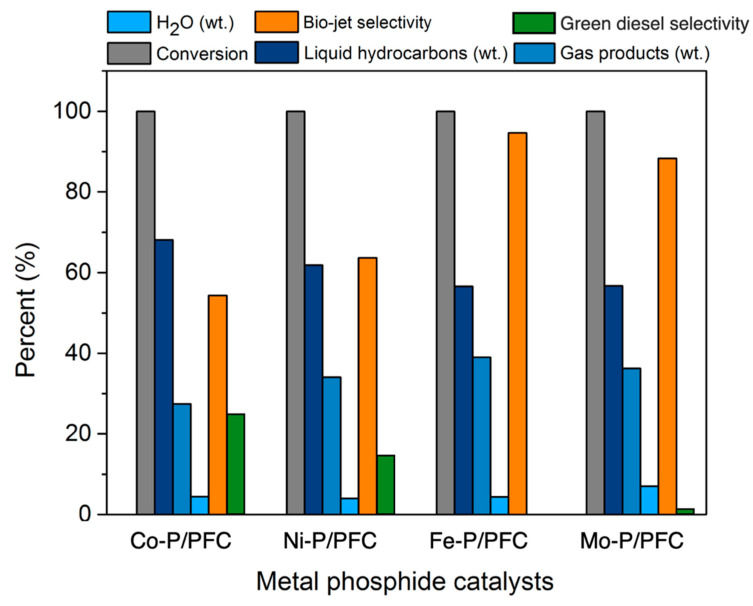
Product contribution of palm olein oil hydrocracking over different metal phosphide catalysts; conversion, byproducts (H_2_O and gas products), product compositions, bio-jet selectivity, and green diesel selectivity.

In this section, the tests of synthesized metal phosphide/PFC catalysts for the palm olein hydrocracking to produce a bio-jet fuel were employed in a stainless-steel trickle bed continuous flow reactor. The effects of different metal phosphide catalysts were investigated in terms of their contribution to liquid hydrocarbon products. The experimental condition was fixed at a reaction temperature of 420 °C, H_2_ pressure of 50 bar, the H_2_ flow rate of 100 mL/min, and LHSV of 1 h^−1^, respectively. Before the reaction, the relative synthesized catalyst was packed in the middle zone of the reactor and subsequently reduced at 600 °C under a H_2_ flow of 100 cm^3^/min for 3 h before the start of the reaction. Figure 7 shows the product contribution of palm olein oil hydrocracking over different metal phosphide catalysts. Among four metal phosphide catalysts exhibited very high catalyst activity as 100% palm olein oil conversion was obtained (Figure 7). This might be due to the optimum conditions for hydrocracking and the high performance of metal phosphide species in the hydrotreating process [3,4]. However, the highest hydrocracking activity was obtained from Fe-P, followed by Mo-P due to the very small catalyst particle size and high catalyst dispersion. These product composition results revealed that high active catalysts (Fe-P and Mo-P) obtained lower liquid hydrocarbons yield (56.61% and 56.72%) when compared to Co-P and Ni-P (68.11% and 61.93%). In addition, the gas products were significantly increased due to the cracking reaction of long-chain hydrocarbons to short-chain hydrocarbons [38]. This implies that the Fe-P and Mo-P catalysts were more selective for light molecule hydrocarbons as a bio-jet fuel fraction. The highest bio-jet selectivity was obtained from Fe-P and Mo-P at 95.62% and 88.34%, respectively, as illustrated in Figure 7. However, there is no hydrocarbon in the green diesel fraction (C_5_–C_18_ hydrocarbons) obtained from the catalytic test with the supported Fe-P catalyst, while the small amount of green diesel was observed with the Mo-P catalyst yielded approximately 1.87% selectivity. Furthermore, the catalytic deoxygenation of palm oil over the supported Co-P and Ni-P catalysts exhibited about 24.27% and 16.32% green diesel selectivity, respectively. This can imply that the supported Co-P and Ni-P catalysts are insufficient for hydrocracking reactions.

**Figure 8:**
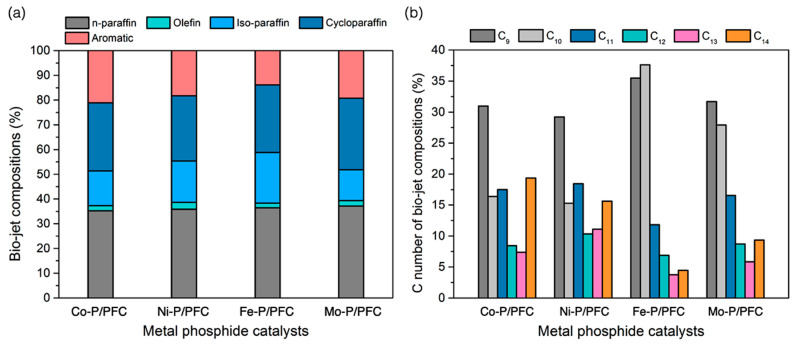
Bio-jet fuel product compositions. (**a**) Bio-jet fuel composition, and (**b**) C number of the bio-jet fuel composition.

In addition, the product compositions of the obtained bio-jet fuel are shown in Figure 8 and separated into two criteria of bio-jet fuel composition based on hydrocarbon structures (Figure 8a) and C number of bio-jet fuel compositions (Figure 8b). Normal paraffin hydrocarbons (n-paraffins), which have a high-quality hydrocarbon structure in fuels, become the desired product in bio-jet fuel fractions [4,38]. The studied catalysts exhibited a high bio-jet in n-paraffins between 35.27 and 37.13%. The highest percentage of n-paraffins was obtained from Fe-P. In addition, a significant reduction of aromatics in the product was obtained from Fe-P (reduced from 21.12% to 13.87%). This refers to the high catalyst performance in the hydrocracking reaction, and it shows the effectiveness for producing high-quality biofuel since the aromatics may increase the cold flow property of biofuel [38,39]. However, the results revealed that the highly active catalyst (Fe-P) tended to promote the isomerization of the short-chain hydrocarbons, indicating a significant increase in iso-paraffin yield. This could be due to the hydrocracking reaction, high acidity, surface area, and catalyst dispersion.

In the reaction pathway, triglycerides and fatty acids in the palm olein oil were transformed into C_15_–C_18_ alkanes through catalytic deoxygenation. Then, the C_15_–C_18_ alkanes were cracked into C_9_–C_14_ alkanes or dehydrogenated to aromatic hydrocarbons. On the other hand, it is directly converted into C_9_–C_14_ alkanes via a cracking reaction from precursors [3,4]. However, the results found that the main hydrocarbon composition obtained from Fe-P and Mo-P, which exhibited greater hydrocracking activity, were C_9_ (35.49% and 31.68%) and C_10_ (31.62% and 27.91%) hydrocarbons. Notwithstanding, a high fraction of long-chain hydrocarbons were obtained from Co-P and Ni-P, which should be attributed to the low acidity leading to lower hydrocracking activity [40].

**Figure 9:**
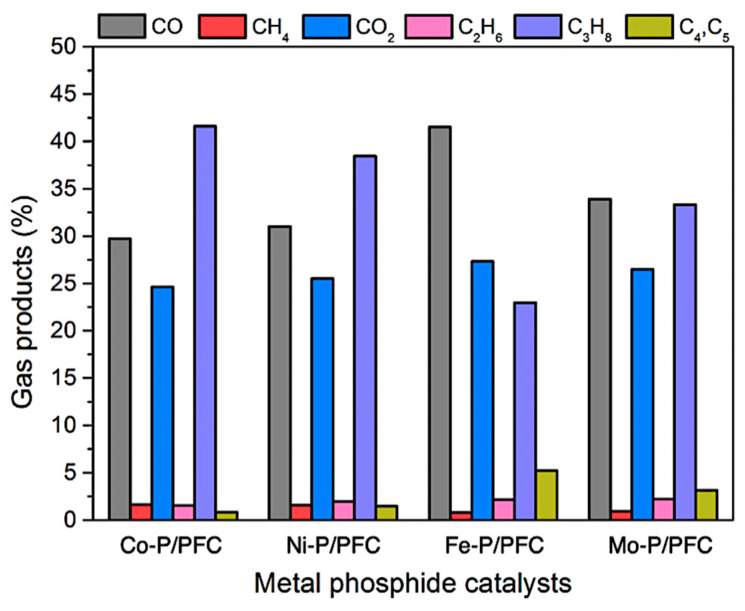
Gas products during palm olein oil hydrocracking over different metal phosphides catalysts.

Moreover, the gas product compositions produced during the hydrocracking of palm olein oil, are displayed in Figure 9. The major gas fraction was released from deoxygenation (i.e., hydrodeoxygenation, decarboxylation, and decarbonylation) and hydrocracking reaction in the form of CO/CO_2_, and gaseous hydrocarbons. Firstly, C_3_H_8_ gas is produced from the hydrogenolysis of saturated triglycerides in the palm olein structure. Gas products can be produced more from the cracking reaction of gaseous hydrocarbons such as C_4_ and C_5_ hydrocarbons [7,8]. However, the CH_4_ and C_2_H_6_ were increased by the simultaneous cracking of C_3_H_8_. The findings revealed that the gas products were mainly composed of CO and CO_2_, indicating that decarboxylation and decarbonylation were the main routes. In the palm olein hydrocracking over the studied metal phosphide catalysts, the highly active catalysts (Fe-P and Mo-P) showed a q high fraction of bio-jet fuel. Thus, it can be confirmed that the Fe-P and Mo-P catalysts exhibited a high proportion of C_4_ and C_5_ gaseous hydrocarbons (Fe-P > Mo-P), a byproduct of the hydrocracking process.

#### 3.2.2. Catalyst Stability Test

**Figure 10:**
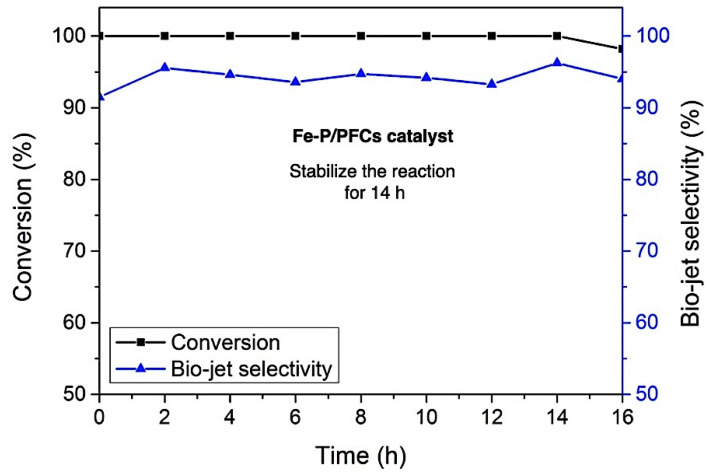
Stability test of Fe-P/PFC catalyst on the palm olein hydrocracking (conversion and bio-jet fuel selectivity). Fe-P/PFC catalyst was selected for the stability test as the effective catalyst to obtain the highest bio-jet fuel selectivity.

The catalyst stability during the reaction was carried out using Fe-P/PMF catalyst, which exhibited the highest bio-jet fuel selectivity without any regeneration treatment under the same reaction conditions (reaction temperature of 420 °C, H_2_ Pressure of 50 bar, and LHSV of 1 h^−1^). In the long-term stability test, deactivation was observed after long-run reactions for the time on the stream of 14 h, which confirmed that Fe-P showed resistance to carbon monoxide, water, and short-chain hydrocarbon poisoning during the reaction, as illustrated in Figure 10. After a continuous period run of 14 h, the catalytic activity decreased as seen in a slight decrease in both palm olein conversion and bio-jet fuel selectivity, suggesting that the catalyst was deactivated due to the coking formation, which generated by light hydrocarbon molecules (the main product) occurred in the hydrocracking during reaction test [41].

## 4. Conclusions

Palm fiber-derived porous biochar (PFC) is an effective support material with a very high surface area. It can be a suitable material for synthesizing active catalysts by wet impregnating different metal phosphide catalysts, referred to as a low-cost material based on its feedstock, high efficiency, and reusability. The characterization of studied catalysts showed the successful impregnation of active metal phosphides into carbon support. The metal phosphide supported on PFC catalysts was successfully utilized for hydrocracking palm olein oil to produce bio-jet fuel. Metal phosphide/PFC catalysts were very active in the hydrocracking of palm olein oil in the following order: Fe-P > Mo-P > Ni-P~Co-P. Moreover, shorter-chain hydrocarbons are the main product for Fe-P/PFC and Mo-P/PFC catalysts, mostly C_9_ and C_12_ hydrocarbons. In contrast, Ni-P and Co-P mainly produced a C_9_ fraction. Fe-P and Mo-P exhibited higher catalyst activity because of their greater acidity (NH_3_-TPD profile) and the excellent active phase dispersion as shown in TEM, XRD, and N_2_ sorption analyses, which, in sum, promote hydrocracking. The Fe-P/PFC catalyst is the most active and selective for palm olein hydrocracking to produce bio-jet fuel. Furthermore, the Fe-P catalyst showed a deactivation after 14 h during the catalyst stability test.

## Figures and Tables

**Table 1:** Textural properties of the support and studied catalysts.

Catalyst	BET Surface Area (m^2^/g)	Total Pore Volume (V_T_, cm^3^/g)	Total H_2_ Consumption (μmol/g)	D_XRD_ (nm)
**PFC Support**	964.04	0.574	n.a.	n.a.
**Ni-P/PFC**	706.72	0.379	231.6	13.38
**Co-P/PFC**	698.54	0.342	193.8	21.66
**Mo-P/PFC**	629.17	0.316	216.5	5.13
**Fe-P/PFC**	792.68	0.427	276.2	4.45

(n.a.) not available.

## Data Availability

Not applicable.
